# Action of Phytochemicals on Insulin Signaling Pathways Accelerating Glucose Transporter (GLUT4) Protein Translocation

**DOI:** 10.3390/molecules23020258

**Published:** 2018-01-28

**Authors:** Abu Sadat Md Sayem, Aditya Arya, Hamed Karimian, Narendiran Krishnasamy, Ameya Ashok Hasamnis, Chowdhury Faiz Hossain

**Affiliations:** 1 Department of Pharmacy, Faculty of Medicine, University of Malaya, 50603 Kuala Lumpur, Malaysia; sayem066@gmail.com; 2 Department of Pharmacology and Therapeutics, School of Medicine, Faculty of Health and Medical Sciences, Taylor’s University, Lakeside Campus, 47500 Subang Jaya, Malaysia; hamedkarimian61@gmail.com (H.K.); AmeyaAshok.Hasamnis@taylors.edu.my (A.A.H.); 3 Clinical Skills, School of Medicine, Faculty of Health and Medical Sciences, Taylor’s University, Lakeside Campus, 47500 Subang Jaya, Malaysia; Narendiran.Krishnasamy@taylors.edu.my; 4 Department of Pharmacy, Faculty of Sciences and Engineering, East West University, Dhaka-1212, Bangladesh; faiz@ewubd.edu

**Keywords:** insulin receptor, insulin signaling pathways, PI3K, APS, GLUT4 translocation, phytochemicals

## Abstract

Diabetes is associated with obesity, generally accompanied by a chronic state of oxidative stress and redox imbalances which are implicated in the progression of micro- and macro-complications like heart disease, stroke, dementia, cancer, kidney failure and blindness. All these complications rise primarily due to consistent high blood glucose levels. Insulin and glucagon help to maintain the homeostasis of glucose and lipids through signaling cascades. Pancreatic hormones stimulate translocation of the glucose transporter isoform 4 (GLUT4) from an intracellular location to the cell surface and facilitate the rapid insulin-dependent storage of glucose in muscle and fat cells. Malfunction in glucose uptake mechanisms, primarily contribute to insulin resistance in type 2 diabetes. Plant secondary metabolites, commonly known as phytochemicals, are reported to have great benefits in the management of type 2 diabetes. The role of phytochemicals and their action on insulin signaling pathways through stimulation of GLUT4 translocation is crucial to understand the pathogenesis of this disease in the management process. This review will summarize the effects of phytochemicals and their action on insulin signaling pathways accelerating GLUT4 translocation based on the current literature.

## 1. Introduction

**Figure 1:**
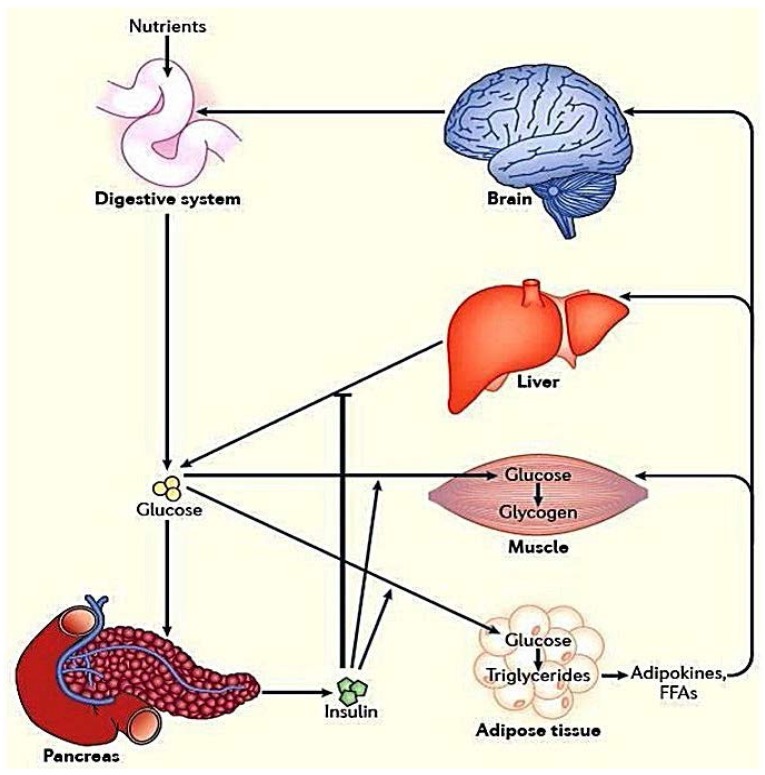
Visualization of the whole-body glucose homeostasis. Glucose homeostasis rely on the function of several organs as well as tissues, including the digestive system, pancreas, brain, liver, muscle and adipose tissue with the help of insulin stimulation, these tissues obtain energy and represent glucose to other parts via the available molecules such as insulin. Flaws in the detecting of the energy homeostasis, and the ability to respond appropriately, result in type 2 diabetes mellitus, major metabolic diseases. Abbreviation; FFAs: free fatty acids.

Glucose is a vital source of energy for all eukaryotic cells. Although all human cells use glucose for their energy production, the brain utilizes 80% of consumed glucose under basal conditions. Initially, energy is supplied by breaking down of endogenous glycogens which are stored in the liver for whole body. Then these storages are replenished by glucose from the diet. Following carbohydrate digestion and glucose absorption in the circulation, it is distributed among the various tissues in the body with the stimulation of insulin secretion (Figure 1) [1]. Effect of this stimulation in transmembrane transport of glucose into extrahepatic tissues was proposed by Goldstein and co-authors in 1949 and was extended by Morgan and co-authors in 1961 [2,3]. Inherent high glucose requirement and rapid glucose-transport system of the brain were reported [1]. Like brain, muscle and adipose tissue have adapted with a highly specialized glucose-transport system for rapid uptake of glucose. This is particularly important during exercise to meet rapidly high metabolic demand of skeletal muscle. This rapid glucose-transport system is also crucial for insulin dependent glucose storage in muscle and adipose tissues after a meal, which assists body to maintain normal blood glucose levels constantly. In 1980, Suzuki and Kono proved that insulin mediates a glucose-transporting activity to the plasma membrane from an intracellular storage site [4,5]. Glucose distribution is performed by a family of glucose transporters (GLUTs) which act as vehicles to move sugar across the cell surface [6,7].

Glucose transporter isoform 1 (GLUT1), the first identified human glucose transporter protein, was cloned in 1985. It is widely expressed and is not markedly compartmentalized by insulin [8]. However, glucose transporter isoform 4 (GLUT4) is the main insulin-responsive glucose transporter and is located predominantly in muscle and fat cells [9]. GLUT4 was cloned and studied in several laboratories in 1989 [10,11]. Those studies showed that GLUT4 proteins are translocated to the plasma membrane by the action of insulin through the insulin signaling pathway. Thus, the physiological concern of how insulin accelerates glucose uptake was transformed into the cell biological concern of how insulin stimulates the GLUT4 membrane trafficking. Insulin enhances glucose uptake potentially by elevating the concentration of GLUT4 proteins at the plasma membrane, rather than by enhancing the intrinsic activity of the transporter [12,13].

The cellular location of GLUT4 is directed by a regulated recycling process, in which endocytosis, sorting into GLUT4 storage vesicles (GSVs), exocytosis, tethering, docking, and fusion of the proteins are all tightly regulated. To understand the mechanism of cellular glucose uptake, two distinct fields critical: (i) Signaling from insulin receptor and (ii) GLUT4 translocation. Not surprisingly, identifying the molecules that correlate insulin signaling to the GLUT4 trafficking has been a major focus of research [14]. By commencing several signaling pathways, insulin controls energy metabolism that regulates cells growth and survival, as well as uptake, synthesis and hydrolysis of glycogen, proteins and lipids [15]. Some of these signaling pathways, including the mammalian target of rapamycin complex 1 (mTORC1) and extracellular signal-regulated kinase (ERK) pathway, are not large enough in controlling of transport of glucose [16,17]. As an alternative, adaptor protein with pleckstrin homology and Src homology 2 (SH2) domain (APS) and a phosphoinositide 3-kinase (PI3K) dependent insulin signaling pathways are required by glucose transport in adipose tissue. Src (abbreviated form of sarcoma) is a proto-oncogene encoding one of tyrosine kinases [18]. Of note, the PI3K dependent insulin signaling pathway for glucose uptake in muscle is fairly well-known, although the necessity for the APS-dependent insulin signaling pathway in this particular tissue is still elusive. All these pathways together, clarify the delivery efficiency of GLUT4 to the cell surface by assembling signaling stage at the plasma membrane that are comprised of protein kinases, lipids, adaptor pro­teins, small GTPases (enzymes that hydrolyze guanosine triphosphate, GTP) and lipid kinases. These signaling pathways involve the compartmentalizing mechanism in regulation of GLUT4 cycling. However, this GLUT4 cycling can also be overstimulated in case of various cancers, like plasma cell malignancy, multiple myeloma, etc. To treat such condition, several studies have been performed to develop GLUT4 antagonist synthetically [19,20]. The insulin hormone is responsible for glucose homeostasis which is released by the pancreatic β-cells [21]. Inappropriate insulin utilization, leading cause of insulin resistance, is a defective condition characterized by the failure of cell response to standard circulating insulin levels [22] Phytochemicals could be possible alternatives in treatment of type 2 diabetes mellitus or supports in current treatment used. They might also be more effective and have less side-effects than the present medications as well as reduce the risks of the disease. One positive aspect is that large amounts of phytochemicals are already consumed in the daily diet. There are plenty of phytochemicals which are discussed in litera­ture based on their effects against insulin resistance or type 2 diabetes. There are many plants which have been used since ancient times in preventing the conditions which are asso­ciated with insulin resistance [23]. Though the possible mechanism is not absolutely understood, numerous studies are being conducted to reveal the different signaling pathways by which various phytochemicals act. The main insulin-responsive GLUT isoform is GLUT4 which is predominantly present in skeletal muscle and adipose tissues. We, therefore, have discussed on the insulin signaling system with GLUT4, the recent understand­ing of how GLUT4 are compartmentalized by insulin signaling pathways and the role of phytochemicals on these signaling pathways in GLUT4 translocation for prophylaxis and treatment of insulin resistance in GLUT4 located tissues.

## 2. Phytochemicals

Medicinal plants have been the basic source of drugs from ancient times and are used extensively as crude drugs for treating various diseases. Some 1–10% of the estimated 250,000 to 500,000 species of plants on Earth are used by humans [24]. Based on the recognized bioactive phytochemicals, polyphenols are the most widespread compounds due to antioxidant and antidiabetic effects of these compounds. The role of polyphenols in glucose homeostasis and carbohydrate metabolism has been well studied and investigated in in-vitro and in-vivo models as well as in some clinical experiments. Some reported antidiabetic effects of phytochemicals through insulin signaling pathways by accelerating GLUT4 translocation are listed in Table 1.

**Figure 2:**
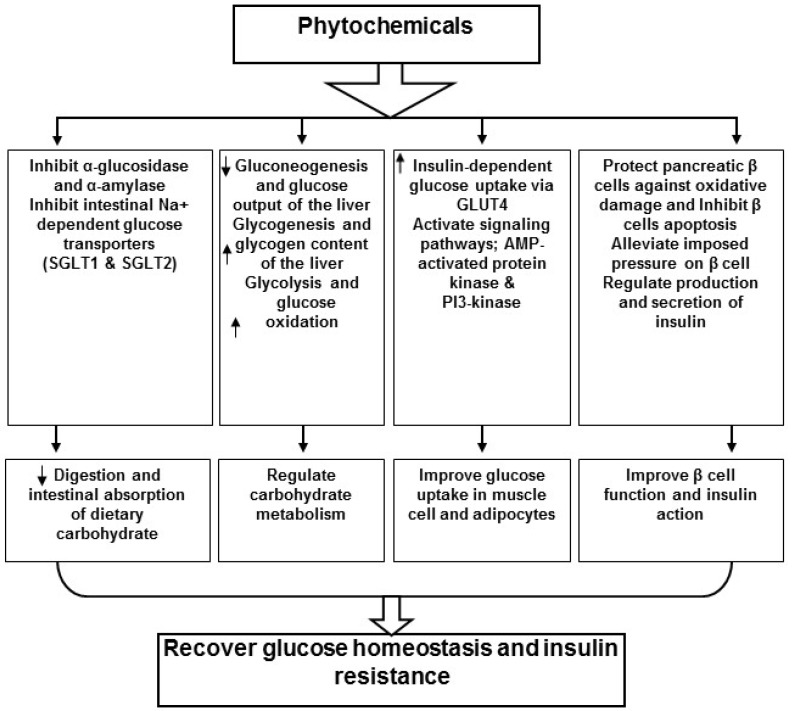
The hypoglycemic effects of phytochemicals are chiefly ascribed to lower the uptake of carbohydrates in intestine affecting the glucose metabolism by applying an alteration in the enzyme activities, β-cell function betterment and insulin action improvement, insulin release initiation and antioxidant as well as anti-inflammatory characteristic of these components. SGLT: Sodium-Glucose Linked Transporter.

The hypoglycemic effects of polyphenols are chiefly ascribed to lowering the uptake of carbohydrates in the intestine, affecting the glucose metabolism by altering enzyme activities, improving β-cell function and insulin action, initiating insulin release and antioxidant as well as anti-inflammatory effects. [35,36,37]. The impacts of the naturally occurring components on the expression of genes are ongoing intensive research to stipulate the mechanisms and novel targets by which these active constituents act as anti-diabetic. Polyphenolic phytochemicals may stimulate gene expressions involved in the occurrence of type 2 diabetes, such as genes controlling the transportation of glucose, release of insulin and its function, antioxidant effect, inflammation, vascular functions, lipid metabolism and thermogenic or other probable mechanisms [38,25,39]. Several phytochemicals have been investigated in diabetic rodent models to disclose gene expression data, insulin signaling pathways and glucose transporters data of different target tissues. Some phytochemicals play an important role in insulin signaling pathways and their impact on GLUT4 in regulating glucose uptake. For instance, resveratrol has been observed to induce AKT (also known as protein kinase B) and VEGF (vascular endothelial growth factor) in streptozotocin (STZ)-induced diabetic rat myocardium compared to non-diabetic animals as well as to increase the expression of GLUT4 in muscle of STZ-induced diabetic rats via PI3K-AKT pathways. Pathologically, lower GLUT4 induction was found in diabetic’s state [25] Valuable effects of polyphenols on maintenance of plasma glucose in diabetics are summarized in Figure 2.

### GLUT Proteins: Structure and Function

The GLUTs (or SLC2A), member of the solute carrier family, are membrane transport proteins that facilitate glucose transportation over plasma membrane, composed of more than five thousand members in all three kingdoms [40,41]. The presence of these transporters in all phyla suggests that glucose is a vital source of energy for all forms of life. Many people believe that there are 14 GLUTs in humans [9] and these 14 isoforms are classified into class I (GLUTs 1–4, 14), class II (GLUTs 5, 7, 9 and 11) and class III (GLUTs 6, 8, 19, 12 and 13). GLUTs are the main regulators of glucose homeostasis in the body and every cell type of the body consists of at least one GLUT isoform. In order to insulin dependent glucose transport, GLUT4 isoform mainly needs to be translocated from an intracellular site to the plasma membrane to enhance glucose uptake. Therefore, GLUT4 is responsible for insulin-stimulated glucose uptake [42,43]. This transporter plays a major role in the maintenance of plasma glucose, since it is primarily expressed in tissues associated with insulin mediated glucose uptake, such as skeletal muscle and adipose tissues [43]. Since GLUT4 is dependent on insulin for the uptake of glucose and its impairment can cause insulin resistance and the aim of this review is to discuss the insulin signaling pathway, more emphasis has been given on this glucose transporter protein. In Table 2, reported features of GLUT4 including others of class I glucose transporters are described.

## 3. GLUT-4 Targets Insulin Signaling Pathway

Insulin signaling coordinates GLUT4 mobilization from intra­cellular membrane particles, GSVs recognition at plasma membrane, at finally these two membranes are fused through multiple engaging of GTPases which start cycling between an ‘active’ GTP-bound conformation and they facilitate an ‘inactive’ guanosine diphosphate (GDP)-bound state and their biological effects. Multiple components of the compartmentalizing machinery interact with active GTPases to confer alignment and specificity in membrane flow [46]. Furthermore to small GTPases, insulin signaling directly targets motor proteins, fusion-regulating proteins and membrane tethers which propose the insulin conducts several phases in the GLUT4 trafficking route to elevate the concentration of the transporter on the cell surface. The insulin signaling pathways increase the levels of GLUT4 of plasma membrane mostly by raising exocytosis of GSVs. Yet, studies show insulin might also affect the endocytosis, sorting and formation of GSVs in the GLUT4 membrane trafficking [47,48].

### 3.1. Insulin Signaling Pathway and GLUT4 Trafficking

**Figure 3:**
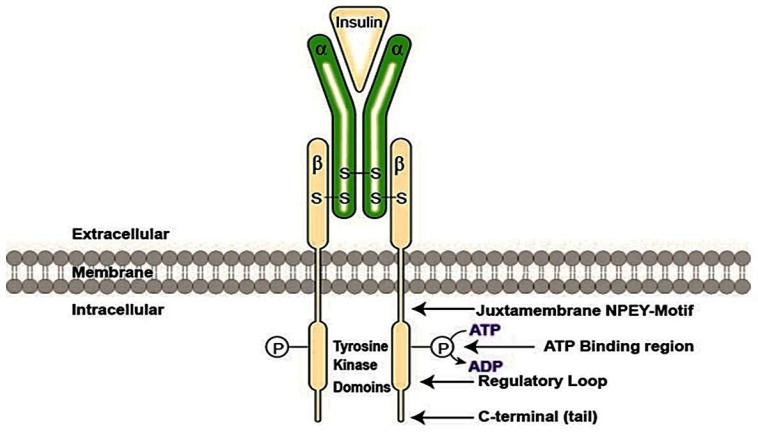
Insulin activates insulin receptor. Subjection of insulin to its receptor brings in the action of insulin and activates insulin receptor which is a type of tyrosine kinase and is comprised of two extracellular α subunits and two cytoplasmic β subunits. Attachment of α subunits to the insulin hormone induces the phosphorylation of β subunits and transferring signals in the entire membrane and activates the intracellular tyrosine kinase domain of the β subunit. Insulin binding activates the tyrosine kinase which leads to autophosphorylation of tyrosine residues in several regions of the intracellular β-subunit, including juxtamembrane region, regulatory loop and C-terminal. The activated IR then phosphorylates phosphotyrosine binding domains on intracellular substrates. Abbreviations: ATP-Adenosine triphosphate, ADP- Adenosine diphosphate, IR- Insulin Receptor, NPEY; Asn-Pro-Glu-Tyr-Motif.

Insulin express its action upon its connection to insulin receptor (IR), which is a type of tyrosine kinase comprised of two extracellular α subunits considered as insulin binding sites and two cytoplasmic β subunits. Attachment of α subunits to the insulin hormone induces the phosphorylation of β subunits and transferring signals in the entire membrane. This event activates the intracellular tyrosine kinase domain of the β subunit [13] leading to auto-phosphorylation of tyrosine residues in several regions of this subunit, including juxtamembrane region, regulatory loop and C-terminal (Figure 3) [49,50].

Then, activated IR phosphorylates phosphotyrosine binding domains on the intracellular substrates, including insulin receptor substrate (IRS) family, Gab-1, CBL, APS and Shc (adapter protein) isoforms and signal regulatory protein (SIRP) family members [13,51]. Linkage of insulin to its receptor which induces tyrosine kinase operation as a key factor involved in the insulin activity. Mutations in the Adenosine triphosphate (ATP) binding domain abolish ATP binding, resulting in shutdown of kinase activity and insulin signaling [52].

### 3.2. Activation of PI3K Pathway

**Figure 4:**
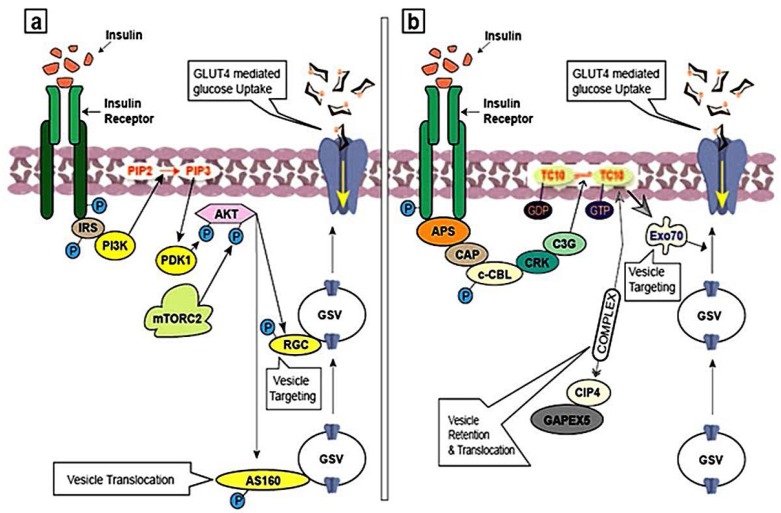
Insulin signaling cascades regulate GLUT4 trafficking by intracellular itinerary. (**a**) PI3K-mediated insulin signaling pathway: Being active by insulin binding, this pathway leads to the involvement and phosphorylation of IRS adaptor proteins. Tyrosine phosphorylated IRS proteins bind with PI3K. The interaction of IRS proteins and PI3K consequences in the activation of PI3K and this catalyzes the successive synthesis of PIP_3_ from PIP_2_ at the plasma membrane. PIP_3_ in turn acts as a docking site for several pleckstrin homology (PH) domain-containing Serine/threonine kinases that are implicated in glucose uptake, including phosphoinositide-dependent kinase 1 (PDK1) and AKT. Through dual serine/threonine phosphorylation, PDK1 and mammalian target of rapamycin 2 (mTORC2) activate AKT. AKT then stimulates the small GTPases, AS160 and the RAL-GAP complex (RGC) which are involved in GLUT4 vesicle translocation, directed to the plasma membrane. (**b**) APS-dependent insulin signaling pathway: Insulin binding also initiates an APS-dependent insulin signaling cascades by recruiting and tyrosine phosphorylation of the adaptor protein APS. Upon IR phosphorylation, APS recruits a complex that includes c-CBL (protein encoded by *CBL* gene) and catabolite activator protein (CAP). This causes insulin receptor-catalyzed tyrosine phosphorylation of c-CBL. Phosphorylated c-CBL then activates the adaptor protein CRK, which is in intricate with C_3_G which is a guanine nucleotide exchange factor (GEF). C_3_G in turn activates TC_10_ which is originated in lipid rafts in the plasma membrane. Active TC_10_ (a member of small GTP-binding proteins) regulates GLUT4 vesicle exocytosis by interacting with several effector proteins. One TC_10_ binding protein, CIP4, recruits a stable complex with the RAB GEF GAPEX5. This regulates GLUT4 vesicles retention and translocation. Another EXO_70_ (a subunit of exorcist complex), TC_10_ effector protein and a subunit of the exocyst tethering complex, has been implicated in GLUT4 vesicle targeting.

Binding of insulin to IR activates the PI3K-mediated insulin signaling pathway, which involves and phosphorylates IRS proteins [15]. Afterwards, IRS molecules build a connection with p85 regulatory subunit of I PI3K class by acting as docking sites for the SH2 domain. Activity of IRS molecules together with PI3K activates and the successive formation of phosphatidylinositol-3,4,5-trisphosphate (PIP_3_) from phosphatidylinositol-4,5-bisphosphate (PIP_2_) at the plasma membrane (Figure 4a). In turn, PIP_3_ acts as a docking site for several PH domain-containing serine/threonine kinases which are associated in glucose uptake, including phosphoinositide-dependent kinase 1 (PDK1) and AKT [53]. Through dual serine/threonine phosphorylation, PDK1 and mTORC2 activate AKT. The mostly studied targets of AKT are the RAB GAP AS160 and RAL-GAP complex (RGC), belongs to the family of small GTPases. These small GTPases are involved in GLUT4 vesicle translocation, directed to the plasma membrane [54,55]. Synip and CDP138, categorized as the soluble N-ethylmaleimide-sensitive factor attachment protein receptor (SNARE) regulatory proteins, are direct substrates for AKT and regulate GLUT4 vesicle fusion with the plasma membrane [47,56].

Studies in the presence of IRS/AKT inhibitors or dominant-negative IRS/AKT have revealed the necessity of IRS molecules in addition to AKT in the absorption of glucose [57,58]. The active AKT over expression can significantly but not entirely emulate insulin efficiency [59,60]. AKT, a focal axis links insulin signals to the modulator proteins of GLUT4 trafficking. Molecular studies that investigated GLUT4 trafficking in the knockout or knockdown of AKT constructs demonstrated that kinase affects the exocytic domain of the GLUT4 trafficking itinerary, by targeting and fusion of GLUT4 containing vesicles and regulating the translocation [54,61]. The experimentation for AKT targets in muscle and adipocytes continue, probably gives further intuition into the definite phases of GLUT4 trafficking which is regulating by this kinase.

### 3.3. Activation of APS Pathway

Insulin binding to the activated insulin receptor initiates an APS-dependent insulin signaling cascades by recruiting and tyrosine phosphorylation of the adaptor protein APS [62]. Upon the addition of phosphate group to IR, APS activate a complex following the c-CBL-associated protein (CAP) and activation of proto-oncogene c-CBL [63,64]. This causes IR-catalyzed tyrosine phosphorylation of c-CBL which in turn activates signal transduction molecules such as CRK, which is in involved with the guanine nucleotide exchange factor (GEF) protein cyanidin 3-glucoside (C_3_G) (Figure 4b) [65]. C_3_G then activates TC_10_, a member of the RHO-family of small GTPases [66,67] originated from lipid rafts in the plasma membrane. Active TC_10_ regulates GLUT4 vesicle exocytosis by interacting with several effector proteins. One TC_10_ binding protein, CDC42 interacting protein 4 (CIP4), recruits a constant complex with the RAB GEF GAPEX5. This regulates the activity of RAB5 family GTPases, which are involved in GLUT4 vesicles retention and translocation. Another EXO_70_, TC_10_ effector protein and a subunit of the exocyst tethering complex, has been implicated in GLUT4 vesicle targeting [47].

Disrupting individual components of the PI3K-mediated or the APS-mediated insulin signaling pathways in adipocytes, either by proteins inhibitors or with small interfering RNA (siRNA)-mediated knockdown which inhibits glucose uptake and GLUT4 exocytosis, [47,57,68] suggests that these pathways control the GLUT4 trafficking machinery. The PI3K signaling pathway is important for GLUT4 exocytosis in muscle cells. The need for an APS signaling route in the absorption of glucose upon the stimulation by insulin is uncertain due to conflicting siRNA and mouse knockout studies. In a study, low efficiency of TC_10_α regardless of TC_10_β in 3T3-L1 adipocytes induces a restricted suppression in the glucose intake in the response to insulin release and GLUT4 trafficking [68]. However, in another study, CAP, a protein of APS signaling pathway, seems to be crucial in insulin signaling. CAP is significantly induced during adipocyte differentiation and is regulated transcriptionally by the thiazolidinedione family of insulin-sensitizing peroxisome proliferator-activated receptor gamma (PPAR-γ) agonists [69]. In this line, expression of a dominant-interfering CAP mutant in another study completely blocked both insulin-stimulated glucose uptake and GLUT4 trafficking [51], but the siRNA mediated knockdown of c-CBL and CAP proteins did not exert any substantial effect on insulin-stimulated glucose uptake or GLUT4 trafficking [70,71]. Together, APS signaling pathway plays a relatively controversial role at the different molecular targets in insulin-stimulated GLUT4 trafficking.

## 4. Phytochemicals and Insulin Signaling Pathway

Insulin sensibility in mice with diabetes was improved upon the administration of methylswertianin and bellidifolin phytochemicals [72]. The mechanism of action of these phytochemicals is to increase expression of IR, IRS-1 and PI3K proteins which are involved in the insulin signaling pathways. Moreover, both phytochemicals are capable of decreasing the activity of glucokinase (GK) and increasing glucose-6-phosphatase (G6Pase) activity, stimulating beta cells in the pancreas to release insulin. Similarly, it was demonstrated that administration of two gallotannins has increased the mRNA expression of GLUT4 and PI3K in the L6 cells [26]. The bioactive compound, 3β-taraxerol exhibited its function following by glucose transport and its preservation upon glucose consumption and glycogen formation. This study revealed that triterpenoid activated glucose transport through the translocation of GLUT4, was mediated by PI3K dependent activation of AKT protein [27].

In another study, *Astragalus* polysaccharide improved glucose homeostasis and enhanced insulin sensitivity in skeletal muscle of type 2 diabetic mice. The mechanism underlying improvement of insulin sensitivity by *Astragalus* polysaccharide is to regularize the insulin-stimulated PKB-Ser473 phosphorylation and GLUT4 translocation [28]. Cyanidin-3-*O*-β-glucoside and protocatechuic acid possess insulin-like activity in human adipocytes. These phytochemicals increase glucose uptake by enhancing GLUT4 translocation and adiponectin secretion that causes probable improved activity of PPAR-γ [29]. Daidzein activates adenosine monophosphate-activated protein kinase (AMPK) followed by GLUT4 translocation to plasma membrane of muscle cells and enhances glucose homeostasis in type 2 diabetic mice [30]. Iridoid, catalpol, specioside and verminoside showed substantial GLUT4 excitation in the superficial layer of cell to facilitate intracellular glucose uptake [31]. Gallic acid decreases blood glucose in diabetic rats [32] and also enhances glucose uptake through the compartmentalization of GLUT4 to the plasma membrane in adipocytes, isolated from STZ-treated rats [73]. Besides, it has been found that quercetin decreases the expression of the Na^+^-dependent glucose transporter (SGLT1), inhibiting the intestinal glucose absorption in CaCo-2/15 cell lines [74].

Protein tyrosine phosphatase 1B (PTP1B) is an antagonist of the insulin transduction pathway, moreover, dephosphorylated insulin receptor tyrosine kinase (IRTK) and IRS result in the suppression of insulin transduction pathway. The PTP1B knock-out mice exhibited enhanced insulin sensitivity in muscles and liver [75] which revealed an option for the anti-diabetic function of specific PTP1B inhibitors [76]. Ursolic acid and oleanolic acid were identified as competitive PTP1B inhibitors, indicating higher expression of phosphorylated insulin receptor and glucose adsorption [77,78]. Berberine and vanillic acid significantly improve GLUT4 translocation via AMPK-dependent pathway, whereas arecoline does the same via the PPAR-γ pathway [33] Besides, the fungal metabolite, demethylasterrriquinone-B, could directly stimulate IRTK, AKT and ERK pathways, apply glucotropic effects of insulin with no mitogenic effect exertion [79,80].

Apart from the above bioactive compounds, there are many more phytochemicals other than flavonoids have been reported for their anti-hyperglycemic effects [81,82,83]. Piperine, pipernonaline and dehydropipernonaline are alkaloid compounds extracted from *Piper retrofractum* that trigger activation of AMPK signaling pathway and the PPAR-γ protein [84,85]. Another study showed that ellagitannins, a tannin phytochemical, restrain α-glucosidase enzymes [86]. In addition, thiosugars like kotalanol [87] and salacinol [88], also inhibit α-glucosidase enzymes. This inhibitory effect of α-glucosidase enzymes is sometimes analogous to the inhibitory effect of standard drugs, acarbose and voglibose [87,88]. Another anti-hyperglycemic plant biomolecule, mangiferin, is a xanthone compound. In the *Salacia oblonga* extract, the presence of mangiferin enhanced the GLUT4 proteins expression and translocation of this glucose transporter to the surface of L6-myocytes and 3T3-L1 adipocytes, consequently, increasing glucose uptake by the cells [34].

## 5. Conclusions

Understanding the cellular substrates of insulin signaling pathways and their effect on GLUT4 compartmentalization supports the intracellular itinerary of glucose uptake by predominantly GLUT4-containing tissues in both normal and diabetic condition and the role of phytochemicals. Therapeutically, the glucose transporter isoform GLUT4 could be a crucial target to treat insulin resistance, since GLUT4 is an insulin-dependent isoform which is responsible for most insulin-stimulated glucose uptake. Naturally occurring bioactive compounds such as phytochemicals, known as cures for insulin resistance and suppression of the disease, need more research attention. A number of phytochemi­cals evaluated for their antidiabetic properties in insulin signaling pathways and GLUT4 protein have been discussed and reviewed here. Some of these phytochemi­cals display very significant effects. Dietary consumption of bioactive natural compounds reduces the risk of insulin resistance due to GLUT4 impairment in skeletal muscle and adipose tissues. Additionally, phytochemical therapy perhaps offers a new therapeutic approach in pharmacological studies to cure type 2 diabetes or facilitate the greater effect of current treatments.

## Figures and Tables

**Table 1:** Some reported antidiabetic effects of phytochemicals on insulin signaling pathways accelerating glucose transporter isoform 4 (GLUT4).

Phytochemicals	Plants	Effects on Insulin Signaling Pathways Accelerating GLUT4	References
Resveratrol	Numerous plants	Induces AKT and VEGF as well as the expression of GLUT4 in muscle of STZ-induced diabetic rats via PI3K-AKT pathways	[25]
Gallotannins	*Capparis moon*	Increases GLUT4 and PI3K mRNA expression in the L6 cells	[26]
3β-taraxerol	*Mangifera indica*	Activates glucose transport through the translocation of GLUT4, mediating by PI3K dependent activation of AKT protein	[27]
Astragalus polysaccharide	*Astragalus membranaceus*	Regularizes insulin-stimulated PKB-Ser473 phosphorylation and GLUT4 translocation	[28]
Cyanidin-3-*O*-β-glucoside and protocatechuic acid	Numerous plants	Insulin-like activity enhancing GLUT4 translocation and adiponectin secretion	[29]
Daidzein	*Glycine max*	Activates AMPK followed by GLUT4 translocation and enhances glucose homeostasis	[30]
Iridoid, catalpol, specioside and verminoside	*Kigelia pinnata*	Stimulation of GLUT4 trafficking to cell surface	[31]
Gallic acid	*Myriophyllum spicatum*	Decreases blood glucose and also enhances glucose uptake through the compartmentalization of GLUT4 to the plasma membrane	[32]
Berberine and vanillic acid	*Berberis aristata* and numerous plants	Improves the translocation of GLUT4 via AMPK-dependent pathway	[33]
Mangiferin	*Salacia oblonga*	Enhances the GLUT4 proteins expression and translocation of this glucose transporter to the surface of L6-myocites and 3T3-adipocytes	[34]

Abbreviations: AKT: Protein kinase B (also known as PKB), VEGF: Vascular endothelial growth factor, STZ: Streptozotocin, PI3K: Phosphoinositide 3-kinase, AMPK: 5' adenosine monophosphate-activated protein kinase.

**Table 2:** Characteristics of class I glucose transporters (GLUTs 1–4) [9,44,45].

Isoform	Gene	Chromosome	Tissue Distribution	Substrate	Function
GLUT1	*SLC2A1*	1p34.2	Ubiquitous, erythrocytes	Glucose, galactose, dehydroacetic acid	Glucose uptake of tissues, glucose transportation through blood barriers into tissues
GLUT2	*SLC2A2*	3q26.2	Brain, intestine, liver, beta-cells, kidney	Glucose, fructose, galactose, dehydroacetic acid, glucosamine	Facilitated transporter of glucose and fructose, low attraction with large capacity
GLUT3	*SLC2A3*	12p13.31	Brain	Glucose, galactose, dehydroacetic acid	Neuronal uptake, facilitated transporter of glucose with strong affinity
GLUT4	*SLC2A4*	17p13	Skeletal and heart muscles, adipocytes	Glucose, dehydroacetic acid	Insulin dependent transporters, responsible for insulin resistance

## References

[1] Ahn M.Y., Katsanakis K.D., Bheda F., Pillay T.S. (2004). Primary and essential role of the adaptor protein APS for recruitment of both c-Cbl and its associated protein CAP in insulin signaling. J. Biol. Chem..

[2] Goldstein M., Levine R., Klein S., Huddlestun B. (1949). The action of insulin on the distribution of galactose in eviscerated nephrectomized dogs. J. Biol. Chem..

[3] Morgan H.E., Henderson M.J., Regen D.M., Park C.R. (1961). Regulation of glucose uptake in muscle. I. The effects of insulin and anoxia on glucose transport and phosphorylation in the isolated, perfused heart of normal rats. J. Biol. Chem..

[4] Suzuki K., Kono T. (1980). Evidence that insulin causes translocation of glucose transport activity to the plasma membrane from an intracellular storage site. Proc. Natl. Acad. Sci. USA.

[5] Cushman S.W., Wardzala L.J. (1980). Potential mechanism of insulin action on glucose transport in the isolated rat adipose cell. Apparent translocation of intracellular transport systems to the plasma membrane. J. Biol. Chem..

[6] Joost H.G., Bell G.I., Best J.D., Birnbaum M.J., Charron M.J., Chen Y.T., Doege H., James D.E., Lodish H.F., Moley K.H. (2002). Nomenclature of the GLUT/SLC2A family of sugar/polyol transport facilitators. Am. J. Physiol. Endocrinol. Metab..

[7] Bouche C., Serdy S., Kahn C.R., Goldfine A.B. (2004). The cellular fate of glucose and its relevance in type 2 diabetes. Endocr. Rev..

[8] Mueckler M., Caruso C., Baldwin S.A., Panico M., Blench I. (1985). Sequence and structure of a human glucose transporter. Science.

[9] Mueckler M., Thorens B. (2013). The SLC2 (GLUT) family of membrane transporters. Mol. Aspects Med..

[10] James D.E., Strube M., Mueckler M. (1989). Molecular cloning and characterization of an insulin-regulatable glucose transporter. Nature.

[11] Birnbaum M.J. (1989). Identification of a novel gene encoding an insulin-responsive glucose transporter protein. Cell.

[12] Furtado L.M., Somwar R., Sweeney G., Niu W., Klip A. (2002). Activation of the glucose transporter GLUT4 by insulin. Biochem. Cell Biol..

[13] Watson R.T., Kanzaki M., Pessin J.E. (2004). Regulated membrane trafficking of the insulin-responsive glucose transporter 4 in adipocytes. Endocr. Rev..

[14] Simpson F., Jonathan P.W., David E.J. (2001). GLUT4—At the cross roads between membrane trafficking and signal transduction. Traffic.

[15] Saltiel A.R., Kahn C.R. (2001). Insulin signaling and the regulation of glucose and lipid metabolism. Nature.

[16] Lazar D.F., Wiese R.J., Brady M.J., Mastick C.C., Waters S.B., Yamauchi K., Pessin J.E., Cuatrecasas P., Saltiel A.R. (1995). Mitogen-activated protein kinase kinase inhibition does not block the stimulation of glucose utilization by insulin. J. Biol. Chem..

[17] Polak P., Cybulski N., Feige J.N., Auwerx J., Rüegg M.A., Hall M.N. (2008). Adipose-specific knockout of raptor results in lean mice with enhanced mitochondrial respiration. Cell Metab..

[18] Larsen C.J. (1989). The Nobel Prize in physiology and medicine 1989. J. Michael Bishop and Harold E. Varmus. Pathol. Biol. (Paris).

[19] Mishra R.K., Wei C., Hresko R.C., Bajpai R., Heitmeier M., Matulis S.M., Nooka A.K., Rosen S.T., Hruz P.W., Schiltz G.E. (2015). In Silico Modeling-based Identification of Glucose Transporter 4 (GLUT4)-selective Inhibitors for Cancer Therapy. J. Biol. Chem..

[20] Wei C., Bajpai R., Sharma H., Heitmeier M., Jain A.D., Matulis S.M., Nooka A.K., Mishra R.K., Hruz P.W., Schiltz G.E. (2017). Development of GLUT4-selective antagonists for multiple myeloma therapy. Eur. J. Med. Chem..

[21] Sesti G. (2006). Pathophysiology of insulin resistance. Best Practice. Res. Clin. Endocrinol. Metab..

[22] Berg J.M., Tymoczko J.L., Stryer L. (2002). Biochemistry.

[23] Soumyanath A. (2005). Traditional Medicines for Modern Times—Antidiabetic Plants.

[24] Borris R.P. (1996). Natural products research: Perspectives from a major pharmaceutical company. J. Ethnopharmacol..

[25] Das U.N. (1999). GLUT-4, tumor necrosis factor, essential fatty acids and daf-genes and their role in insulin resistance and non-insulin dependent diabetes mellitus. Prostaglandins Leukot. Essent. Fat. Acids.

[26] Kanaujia A., Duggar R., Pannakal S.T., Yadav S.S., Katiyar C.K., Bansal V., Anand S., Sujatha S., Lakshmi B.S. (2010). Insulinomimetic activity of two new gallotannins from the fruits of *Capparis moonii*. Bioorg. Med. Chem..

[27] Sangeetha K.N., Sujatha S., Muthusamy V.S., Anand S., Nithya N., Velmurugan D., Balakrishnan A., Lakshmi B.S. (2010). 3β-taraxerol of *Mangifera indica*, a PI3K dependent dual activator of glucose transport and glycogen synthesis in 3T3-L1 adipocytes. Biochim. Biophys. Acta.

[28] Liu M., Wu K., Mao X., Wu Y., Ouyang J. (2010). Astragalus polysaccharide improves insulin sensitivity in KKAy mice: Regulation of PKB/GLUT4 signaling in skeletal muscle. J. Ethnopharmacol..

[29] Scazzocchio B., Varì R., Filesi C., D’Archivio M., Santangelo C., Giovannini C., Iacovelli A., Silecchia G., Volti G., Galvano F. (2011). Cyanidin-3-O-b-Glucoside and Protocatechuic Acid Exert Insulin-Like Effects by Upregulating PPARg Activity in Human Omental Adipocytes. Diabetes.

[30] Cheong S.H., Furuhashi K., Ito K., Nagaoka M., Yonezawa T., Miura Y., Yagasaki K. (2014). Daidzein promotes glucose uptake through glucose transporter 4 translocation to plasma membrane in L6 myocytes and improves glucose homeostasis in Type 2 diabetic model mice. J. Nutr. Biochem..

[31] Khan M.F., Dixit P., Jaiswal N., Tamrakar A.K., Srivastava A.K., Maurya R. (2012). Chemical constituents of *Kigelia pinnata* twigs and their GLUT4 translocation modulatory effect in skeletal muscle cells. Fitoterapia.

[32] Latha R.C.R., Daisy P. (2011). Insulin-secretagogue, antihyperlipidemic and other protective effects of gallic acid isolated from *Terminalia bellerica* Roxb. in streptozotocin-induced diabetic rats. Chem. Biol. Interact..

[33] Prabhakar P.K., Doble M. (2011). Effect of Natural Products on Commercial Oral Antidiabetic Drugs in Enhancing 2-Deoxyglucose Uptake by 3T3-L1 Adipocytes. Ther. Adv. Endocrinol. Metab..

[34] Girón M.D., Sevillano N., Salto R., Haidour A., Manzano M., Jiménez M.L., Rueda R., López-Pedrosa J.M. (2009). *Salacia oblonga* extract increases glucose transporter 4-mediated glucose uptake in L6 rat myotubes: Role of mangiferin. Clin. Nutr..

[35] Iwai K., Kim M.Y., Onodera A., Matsue H. (2006). α-Glucosidase inhibitory and antihyperglycemic effects of polyphenols in the fruit of *Viburnum dilatatum* Thunb. Agric. Food Chem..

[36] Iwai K. (2008). Antidiabetic and antioxidant effects of polyphenols in brown alga *Ecklonia stolonifera* in genetically diabetic KK-A^y^ mice. Plant Foods Hum. Nutr..

[37] Cabrera C., Artacho R., Giménez R. (2006). Beneficial effects of green tea—A review. J. Am. Coll. Nutr..

[38] Thirunavukkarasu M., Penumathsa S.V., Koneru S., Juhasz B., Zhan L., Otani H., Bagchi D., Das D.K., Maulik N. (2007). Resveratrol alleviates cardiac dysfunction in streptozotocin-induced diabetes: Role of nitric oxide, thioredoxin, and heme oxygenase. Free Radic. Biol. Med..

[39] Moskaug J.Ø., Carlsen H., Myhrstad M., Blomhoff R. (2004). Molecular imaging of the biological effects of quercetin and quercetin-rich foods. Mech. Ageing Dev..

[40] Thorens B., Mueckler M. (2010). Glucose transporters in the 21st Century. Am. J. Physiol. Endocrinol. Metab..

[41] Pao S.S., Paulsen I.T., Saier M.H. (1998). Major facilitator superfamily. Microbiol. Mol. Biol. Rev..

[42] Bryant N.J., Goovers R., James D.E. (2002). Regulated transport of the glucose transporter GLUT4. Nat. Rev. Mol. Cell Biol..

[43] Zisman A., Peroni O.D., Abel E.D., Michael M.D., Mauvais-Jarvis F., Lowell B.B., Wojtaszewski J.F., Hirshman M.F., Virkamaki A., Goodyear L.J. (2000). Targeted disruption of the glucose transporter 4 selectively in muscle causes insulin resistance and glucose intolerance. Nat. Med..

[44] Zhao F.Q., Keating A.F. (2007). Expression and regulation of glucose transporters in the bovine mammary gland. J. Dairy Sci..

[45] Fujita H., Hatakeyama H., Watanabe T.M., Sato M., Higuchi H., Kanzaki M. (2010). Identification of three distinct functional sites of insulin-mediated GLUT4 trafficking in adipocytes using quantitative single molecule imaging. Mol. Biol. Cell.

[46] Cai H., Reinisch K., Ferro-Novick S. (2007). Coats, tethers, Rabs, and SNAREs work together to mediate the intracellular destination of a transport vesicle. Dev. Cell.

[47] Leto D., Saltiel A.R. (2012). Regulation of glucose transport by insulin: Traffic control of GLUT4. Nat. Rev. Mol. Cell Biol..

[48] Nolan J.H., Jeffrey S.E. (2011). Signaling, cytoskeletal and membrane mechanisms regulating GLUT4 exocytosis. Trends Endocrinol. Metab..

[49] Feener E.P., Backer J.M., King G.L., Wilden P.A., Sun X.J., Kahn C.R., White M.F. (1993). Insulin stimulates serine and tyrosine phosphorylation in the juxtamembrane region of the insulin receptor. J. Biol. Chem..

[50] White M.F., Kahn C.R. (1994). The insulin signaling system. J. Biol. Chem..

[51] Saltiel A.R., Pessin J.E. (2003). Insulin signaling in microdomains of the plasma membrane. Traffic.

[52] McClain D.A., Maegawa H., Lee J., Dull T.J., Ulrich A., Olefsky J.M. (1987). A mutant insulin receptor with defective tyrosine kinase displays no biological activity and does not undergo endocytosis. J. Biol. Chem..

[53] Whiteman E.L., Cho H., Birnbaun M.J. (2002). Role of Akt/protein kinase B in metabolism. Trends Endocrinol. Metab..

[54] Gonzalez E., McGraw T.E. (2006). Insulin signaling diverges into Akt-dependent and -independent signals to regulate the recruitment/docking and the fusion of GLUT4 vesicles to the plasma membrane. Mol. Biol. Cell.

[55] Chen X.W., Leto D., Xiong T., Yu G., Cheng A., Decker S., Saltiel A.R. (2011). A Ral GAP complex links PI 3-kinase/Akt signaling to RalA activation in insulin action. Mol. Biol. Cell.

[56] Min J., Okada S., Kanzaki M., Elmendorf J.S., Coker K.J., Ceresa B.P., Syu L.J., Noda Y., Saltiel A.R., Pessin J.E. (1999). Synip: A novel insulin-regulated syntaxin 4-binding protein mediating GLUT4 translocation in adipocytes. Mol. Cell..

[57] Katome T., Obata T., Matsushima R., Masuyama N., Cantley L.C., Gotoh Y., Kishi K., Shiota H., Ebina Y. (2003). Use of RNA interference-mediated gene silencing and adenoviral overexpression to elucidate the roles of AKT/protein kinase B isoforms in insulin actions. J. Biol. Chem..

[58] Clarke J.F., Young P.W., Yonezawa K., Kasuga M., Holman G.D. (1994). Inhibition of the translocation of GLUT1 and GLUT4 in 3T3-L1 cells by the phosphatidylinositol 3-kinase inhibitor, wortmannin. Biochem. J..

[59] Kohn A.D., Summers S.A., Birnbaum M.J., Roth R.A. (1996). Expression of a constitutively active Akt Ser/Thr kinase in 3T3-L1 adipocytes stimulates glucose uptake and glucose transporter 4 translocation. J. Biol. Chem..

[60] Ng Y., Ramm G., Lopez J.A., James D.E. (2008). Rapid activation of Akt2 is sufficient to stimulate GLUT4 translocation in 3T3-L1 adipocytes. Cell Metab..

[61] Bai L., Wang Y., Fan J., Chen Y., Ji W., Qu A., Xu P., James D.E., Xu T. (2007). Dissecting multiple steps of GLUT4 trafficking and identifying the sites of insulin action. Cell Metab..

[62] Yamada E., Okada S., Saito T., Ohshima K., Sato M., Tsuchiya T., Uehara Y., Shimizu H., Mori M. (2005). Akt2 phosphorylates Synip to regulate docking and fusion of GLUT4-containing vesicles. J. Cell Biol..

[63] Ribon V., Printen J.A., Hoffman N.G., Kay B.K., Saltiel A.R. (1998). A novel, multifuntional c-Cbl binding protein in insulin receptor signaling in 3T3-L1 adipocytes. Mol. Cell. Biol..

[64] Liu J., Kimura A., Baumann C.A., Saltiel A.R. (2002). APS facilitates c-Cbl tyrosine phosphorylation and GLUT4 translocation in response to insulin in 3T3-L1 adipocytes. Mol. Cell. Biol..

[65] Ribon V., Hubbell S., Herrera R., Saltiel A.R. (1996). The product of the cbl oncogene forms stable complexes in vivo with endogenous Crk in a tyrosine phosphorylation-dependent manner. Mol. Cell. Biol..

[66] Knudsen B.S., Feller S.M., Hanafusa H. (1994). Four proline-rich sequences of the guanine-nucleotide exchange factor C3G bind with unique specificity to the first Src homology 3 domain of Crk. J. Biol. Chem..

[67] Chiang S.H., Baumann C.A., Kanzaki M., Thurmond D.C., Watson R.T., Neudauer C.L., Macara I.G., Pessin J.E., Saltiel A.R. (2001). Insulin-stimulated GLUT4 translocation requires the CAP-dependent activation of TC_10_. Nature.

[68] Chang L., Chiang S.H., Saltiel A.R. (2007). TC_10_α is required for insulin-stimulated glucose uptake in adipocytes. Endocrinology.

[69] Ribon V., Johnson J.H., Camp H.S., Saltiel A.R. (1998). Thiazolidinediones and insulin resistance: Peroxisome proliferator activated receptor gamma activation stimulates expression of the *CAP* gene. Proc. Natl. Acad. Sci. USA.

[70] Mitra P., Zheng X., Czech M.P. (2004). RNAi-based analysis of CAP, Cbl, and CrkII function in the regulation of GLUT4 by insulin. J. Biol. Chem..

[71] Zhou Q.Á., Park J.G., Jiang Z.Y., Holik J.J., Mitra P., Semiz S., Guilherme A., Powelka A.M., Tang X., Virbasius J. (2004). Analysis of insulin signalling by RNAi-based gene silencing. Biochem. Soc. Trans..

[72] Tian L.Y., Bai X., Chen X.H., Fang J.B., Liu S.H., Chen J.C. (2010). Anti-diabetic effect of methylswertianin and bellidifolin from *Swertia punicea* Hemsl. and its potential mechanism. Phytomedicine.

[73] Naowaboot J., Pannangpetch P., Kukongviriyapan V., Prawan A., Kukongviriyapan U., Itharat A. (2012). Mulberry leaf extract stimulates glucose uptake and GLUT4 translocation in rat adipocytes. Am. J. Chin. Med..

[74] Baldea L.A.N., Martineau L.C., Benhaddou-Andaloussi A., Arnason J.T., Lévy É., Haddad P.S. (2010). Inhibition of intestinal glucose absorption by anti-diabetic medicinal plants derived from the James Bay Cree traditional pharmacopeia. J. Ethnopharmacol..

[75] Elchebly M., Payette P., Michaliszyn E., Cromlish W., Collins S., Loy A.L., Normandin D., Cheng A., Himms-Hagen J., Chan C.C. (1999). Increased insulin sensitivity and obesity resistance in mice lacking the protein tyrosine phosphatase-1B gene. Science.

[76] Arya A., Looi C.Y., Wong W.F., Noordin M.I., Nyamathulla S., Mustafa M.R., Mohd M.A. (2013). In vitro antioxidant, PTP-1B inhibitory effects and in vivo hypoglycemic potential of selected medicinal plants. Int. J. Pharmacol..

[77] Zhang W., Hong D., Zhou Y., Zhang Y., Shen Q., Li J.Y., Hu L.H., Li J. (2006). Ursolic acid and its derivative inhibit protein tyrosine phosphatase 1B, enhancing insulin receptor phosphorylation and stimulating glucose uptake. Biochim. Biophys. Acta.

[78] Zhang Y.N., Zhang W., Hong D., Shi L., Shen Q., Li J.Y., Li J., Hu L.H. (2008). Oleanolic acid and its derivatives: New inhibitor of protein tyrosine phosphatase 1B with cellular activities. Bioorg. Med. Chem..

[79] Zhang B., Salituro G., Szalkowski D., Li Z., Zhang Y., Royo I., Vilella D., Díez M.T., Pelaez F., Ruby C. (1999). Discovery of a small molecule insulin mimetic with antidiabetic activity in mice. Science.

[80] Lin B., Li Z., Park K., Deng L., Pai A., Zhong L., Pirrung M.C., Webster N.J. (2007). Identification of novel orally available small molecule insulin mimetics. J. Pharmacol. Exp. Ther..

[81] Arya A., Al-Obaidi M.M.J., Shahid N., Noordin M.I.B., Looi C.Y., Wong W.F., Khaing S.L., Mustafa M.R. (2014). Synergistic effect of quercetin and quinic acid by alleviating structural degeneration in the liver, kidney and pancreas tissues of STZ-induced diabetic rats: A mechanistic study. Food Chem. Toxicol..

[82] Taha H., Arya A., Paydar M., Looi C.Y., Wong W.F., Murthy C.V., Noordin M.I., Ali H.M., Mustafa A.M., Hadi A.H.A. (2015). Upregulation of insulin secretion and downregulation of pro-inflammatory cytokines, oxidative stress and hyperglycemia in STZ-nicotinamide-induced type 2 diabetic rats by Pseuduvaria monticola bark extract. Food Chem. Toxicol..

[83] Arya A., Al-Obaidi M.M.J., Karim R.B., Taha H., Khan A.K., Shahid N., Sayem A.S., Looi C.Y., Mustafa M.R., Mohd M.A., Ali H.M. (2015). Extract of Woodfordia fruticosa flowers ameliorates hyperglycemia, oxidative stress and improves β-cell function in streptozotocin-nicotinamide induced diabetic rat. J. Ethnopharmacol..

[84] Coman C., Rugina O.D., Socaciu C. (2012). Plants and natural compounds with antidiabetic action. Not. Bot. Horti Agrobot. Cluj-Napoca.

[85] Kim K.J., Lee M.S., Jo K., Hwang J.K. (2011). Piperidine alkaloids from *Piper retrofractum* Vahl. protect against high-fat diet-induced obesity by regulating lipid metabolism and activating AMP-activated protein kinase. Biochem. Biophys. Res. Commun..

[86] Gunawan-Puteri M.D., Kawabata J. (2010). Novel α-glucosidase inhibitors from *Macaranga tanarius* leaves. Food Chem..

[87] Xie W., Tanabe G., Matsuoka K., Amer M.F., Minematsu T., Wu X., Yoshikawa M., Muraoka O. (2011). Role of the side chain stereochemistry in the α-glucosidase inhibitory activity of kotalanol, a potent natural α-glucosidase inhibitor. Bioorg. Med. Chem..

[88] Muraoka O., Morikawa T., Miyake S., Akaki J., Ninomiya K., Yoshikawa M. (2010). Quantitative determination of potent α-glucosidase inhibitors, salacinol and kotalanol, in *Salacia* species using liquid chromatography-mass spectrometry. J. Pharmaceut. Biomed..

